# Breaking barriers - Exploring the Full Cup Test (FCT) pain scale at a tertiary care hospital

**Published:** 2024-12

**Authors:** Nida Ghouri

**Affiliations:** Office of Research, Innovation and Commercialization (ORIC) Indus Hospital & Health Network, Karachi, Pakistan. E-mail: nida.ghouri@tih.org. pknidaaghouri@hotmail.com

Dear Editor,

I recently came across the paper titled “Breaking barriers: Exploring the Full Cup Test (FCT) pain scale at a tertiary care hospital” published in the ICON supplement in 2024. On detailed review of the paper various ambiguities have been observed which raise questions on the credibility of the paper and review process. On the basis of my expertise following major concerns in the paper has been observed;

1. Inappropriate selection criteria. (no exclusion criteria mentioned)
2. On which population study was conducted? adult, pediatrics or elderly?
3. From which ward patients were enrolled? medicine, gynecology or surgery?
4. The calculation performed for FCT is wrong. (As mentioned by ErgÜN U et al reference cited as reference number 19 in the paper, the formula for calculating FCT is length of the line (cm)/height of the cup (cm)×100. However, the author has taken only the height of the cup which makes the result falsified.
5. As analysis seems to be incomplete. In this study reliability analysis is very crucial to evaluate the reproducibility. Author didn’t mention reliability analysis in their study. Furthermore, reliability analysis is also not taken into consideration by the author.
6. There are discrepancies in results mentioned in abstract and main results. For example; median age was 42.5 in abstract while its 44.5 in main results. Similarly, the author mentioned IQR in text while reported range instead of IQR.
7. In abstract, the duration of pain is 4 months and range is 1day to 4 years. However, in Table-I, the duration of pain is 120 months and IQR is 16 to 4 years. Seems ambiguous. Author used multiple units of reporting for one variable which makes the result confusing.

In conclusion, it is requested to the editorial to re-review the paper, as concerns mentioned in point 4 & 5 raise questions on the accuracy of findings.

***Muhammad Imran People’s Primary Healthcare Initiative (PPHI), Karachi, Pakistan. Email: imran.shekhani@hotmail.com***

## Response from the Author:

1. The inclusion and exclusion criteria was not explained in detail due to word count limit. It is as under:
  - Patients who will come in Orthopedics, family medicine, rheumatology outpatient department (OPD) with any acute or chronic pain.
  - Both the gender.
  - Age ≥18 years.
  - Those who understand at least English or Urdu.
    ***Exclusion criteria***
  - Those aged < 18 years and etc.
  - Those who don’t agree to give consent.
2. Our inclusion criteria was only adult ≥18 years
3. At the Indus Hospital, all patients are admitted to the same ward belong to any specialty of department. Our rationale was to enroll only those patients experiencing pain, regardless of the underlying cause.
4. Yes but to simplify the study, we provided a printed image of a glass with a height of 10 cm, allowing for easy and consistent measurement and comparison.
5. We had only a single variable for pain assessment. Therefore, we applied both a correlation and an agreement test. I believe this should not be considered an incomplete analysis.
6. Apology for discrepancy in abstract I don’t how it was not updated the actual descriptive is 44.5 (30-57.5) years with min 18 years and max 78 years of age.
7. Agreed it is confuse If we talk about only one unit which is years so the true result is median (IQR): 0.2 (0.04-4.0) with minute duration one hour and maximum 40 years. (But I again repeat the abstract had some values of 1^st^ analysis but by mistake it was not completed updated. I accept my mistake. But in whole manuscript the results are correct.

I did not agree with the response 4 and 5 given by the author.

***Muhammad Imran***
